# Strategies in Rapid Genetic Diagnostics of Critically Ill Children: Experiences From a Dutch University Hospital

**DOI:** 10.3389/fped.2021.600556

**Published:** 2021-05-31

**Authors:** Miriam E. Imafidon, Birgit Sikkema-Raddatz, Kristin M. Abbott, Martine T. Meems-Veldhuis, Morris A. Swertz, K. Joeri van der Velde, Gea Beunders, Dennis K. Bos, Nine V. A. M. Knoers, Wilhelmina S. Kerstjens-Frederikse, Cleo C. van Diemen

**Affiliations:** ^1^Department of Genetics, University of Groningen, University Medical Centre Groningen, Groningen, Netherlands; ^2^Genomics Coordination Center, University of Groningen, University Medical Center Groningen, Groningen, Netherlands

**Keywords:** neonatal intensive care (unit), next generation sequencing, genetic diagnostics, copy number variation, genetic disease

## Abstract

**Background:** Genetic disorders are a substantial cause of infant morbidity and mortality and are frequently suspected in neonatal intensive care units. Non-specific clinical presentation or limitations to physical examination can result in a plethora of genetic testing techniques, without clear strategies on test ordering. Here, we review our 2-years experiences of rapid genetic testing of NICU patients in order to provide such recommendations.

**Methods:** We retrospectively included all patients admitted to the NICU who received clinical genetic consultation and genetic testing in our University hospital. We documented reasons for referral for genetic consultation, presenting phenotypes, differential diagnoses, genetic testing requested and their outcomes, as well as the consequences of each (rapid) genetic diagnostic approach. We calculated diagnostic yield and turnaround times (TATs).

**Results:** Of 171 included infants that received genetic consultation 140 underwent genetic testing. As a result of testing as first tier, 13/14 patients received a genetic diagnosis from QF-PCR; 14/115 from SNP-array; 12/89 from NGS testing, of whom 4/46 were diagnosed with a small gene panel and 8/43 with a large OMIM-morbid based gene panel. Subsequent secondary or tertiary analysis and/or additional testing resulted in five more diagnoses. TATs ranged from 1 day (QF-PCR) to a median of 14 for NGS and SNP-array testing, with increasing TAT in particular when many consecutive tests were performed. Incidental findings were detected in 5/140 tested patients (3.6%).

**Conclusion:** We recommend implementing a broad NGS gene panel in combination with CNV calling as the first tier of genetic testing for NICU patients given the often unspecific phenotypes of ill infants and the high yield of this large panel.

## Introduction

Approximately 1 in 47 children in the Netherlands is born with one or more congenital malformations, and roughly 25% of these congenital malformations are caused by genetic aberrations (1). A number of these children will require admittance to the neonatal intensive care unit (NICU), where clinical diagnostics may be complicated by a non-specific clinical presentation or limitations to physical examination, especially in the setting of critical clinical conditions. The differential diagnosis process in the NICU is thus often time-consuming, may be burdensome for the patient, and may be ineffective in reaching a genetic diagnosis. The classical genetic diagnostics workflow included molecular and cytogenetic testing to identify aneuploidies and copy number variations and, to a lesser extent, single gene analysis in case of a clear clinical diagnosis. We and others have previously shown that adding rapid next generation sequencing (rNGS) for detection of multiple monogenic diseases adds to the diagnostic yield and lowers the turnaround times (TATs) for diagnoses, thereby improving care for these children and their parents (2–4).

When implementing rNGS, a genetic diagnostic laboratory has to choose a sequencing approach, which can range from small targeted sequencing gene panels, to exome sequencing (ES) using a “virtual” gene panel filter, to whole genome sequencing (WGS) without extensive filtering. Each of these approaches has its benefits and its limitations and a different impact on the laboratory process. While targeted gene panels that investigate a relatively small number of genes can provide a fast TAT, these panels are limited in their range of diagnoses and they cannot be altered easily. On the other side of the spectrum, ES or WGS without stringent filtering may result in a higher diagnostic yield, but their detection of a far larger number of variants generally requires a longer stage of variant interpretation and will result in more incidental findings (5).

In our laboratory at the University Medical Centre Groningen (UMCG), we have now implemented rNGS for NICU patients in two approaches based on parent-child trio ES: the clinical geneticist can request either (1) a dedicated virtual gene panel based on the specific phenotype of the patient, or (2) a broad gene panel (OMIM morbid gene panel) that includes most disease-associated OMIM genes (6) when the patient's phenotype is unspecific and/or unclear. Both approaches can be requested in parallel with SNP-array for copy number variation (CNV) analysis (7), with or without prior QF-PCR for aneuploidy detection. To review and possibly revisit the strategies used in our laboratory, we retrospectively analyzed all the techniques requested for all the patients who received a consultation by a clinical geneticist in the NICU of our University hospital for a period of 2 years. Here, we describe the techniques and approaches chosen, TATs, yields and clinical implications and make recommendations for efficient implementation of rNGS. In addition, we will describe developments that have helped fine-tune our workflows protocols and improve TATs.

## Materials and Methods

### Study Design and Inclusion of Patients

We retrospectively included all patients admitted to the NICU who received clinical genetic consultation and genetic testing in the University Medical Center Groningen (UMCG) between December 2017 and January 2020. Patient records and clinical notes in electronic health records for each patient were accessed to document the reasons for referral for genetic consultation, presenting phenotypes, differential diagnoses, genetic testing requested by the clinical geneticist, reports with results of genetic testing, outcome of cases and post-test counseling information. The data collected were stored on a secure server within the UMCG, and the information was only accessible by those involved in this study. All data were analyzed anonymously. All parents gave informed consent for the gathering of data for research purposes, and the medical ethical board of the UMCG approved the study protocol (waiver number M20.258501).

### Genetic Counseling

Genetic consultations were requested by NICU physicians when a genetic cause was suspected for the congenital malformations and/or abnormalities seen in the patient during admittance. After in-depth physical examination of the patient, parents were counseled about genetic testing in general, risk of incidental findings, risk of uncertain or unclear findings (VUSs) and about the recommended tests for each case. Actionable incidental findings, as defined by the ACMG (8), were reported to the parents. However, parents could opt out of genetic diagnostics at any time and were given the option to not be notified in case of incidental findings. If genetic testing was not deemed to be informative for the observed congenital abnormality, no testing took place and the patient was not included in this study.

### Genetic Diagnostics Workflow

DNA was extracted from peripheral blood of the child and parents using standard diagnostic procedures. QF-PCR (Devyser Complete v2; Devyser) was performed according to standard protocol for trisomies 13, 18, 21, and the X and Y chromosomes (maximum 4 days TAT). Depending on the diagnostic request, routine-care SNP-array (using Global Screening Array (GSA) on an Illumina iSCAN) and ES were either done sequentially, or in parallel, in a patient-parent trio design (maximum 28 days TAT). ES was performed using SureSelect Human All Exon V6 (Agilent, USA) target enrichment, according to standard procedures, on Bravo automated liquid handling robots (Agilent), and then sequenced on an Illumina NextSeq500 sequencer. For reliable results the aim is 20× coverage for 95% of the target genes.

Requested virtual NGS gene panels ranged from phenotype based (PB, ranging from 5 to 329 genes) to a large panel based on all OMIM morbid genes (~4,200 genes); all gene panels used are listed in Supplementary Table 1. In addition to our rNGS diagnostic workflow, a trio-based open-exome analysis of all protein coding genes (~23,000 genes), similar to whole exome sequencing, could also be requested for cases without a diagnosis after rNGS. This test is not offered as rNGS as the interpretation of variants prolongs the TAT.

### Data Analysis

SNP array data was processed in NxClinical/Nexus software (BioDiscovery, Inc., USA). In general, copy number deletions >50 kb containing an OMIM morbid gene, deletions >150 kb, duplications >200 kb and homozygous regions of at least 10 Mb were reported. CNVs seen in 10% or more of our in-house database of healthy controls were considered common (9).

Raw ES data (VCF file) was processed according to standardized protocols, as described previously (10). Sequence variants (single nucleotide variants (SNVs) and small indels) were automatically filtered using Alissa Interpret software (Agilent Technologies, Inc., USA) with the requested virtual gene panel. In general, the filter steps in Alissa included in-house, national and international databases of previously classified variants, the GAVIN (Gene-Aware Variant Interpretation) prioritization tool (11), minor allele frequencies from GnomAD, the disease mode of inheritance reported in OMIM, the Human Gene Mutation Database (HGMD) and OMIM-reported modes of inheritance using MOLGENIS (version 1.4.0) (11–15). Variants that remained after these filtering steps were evaluated by the operating technician, a genetic laboratory specialist and a clinical geneticist in matching the phenotype. A detailed description of the variant-filtering process was published previously (10).

The optional trio-based open-exome analysis examines all protein coding genes in the exome and is performed with an additional filtering step based on a variant's mode of inheritance in the patient (i.e., compound heterozygous variants inherited one from each of the parents). Once filtered, all candidate genes are examined for human disease associations in OMIM and HGMD, and in the citation database of PubMed (NCBI, USA). The literature was also checked for information on relevant animal models.

### Reporting of Variants

All variants that might explain the patient phenotype, and all incidental findings were classified using Alamut software (Interactive BioSoftware/SophiaGenetics) according to standardized guidelines based on Richards et al., while taking into account the HGMD, CADD score and population frequency (9, 16, 17). Variants classified as pathogenic and likely pathogenic were always communicated to parents (8). Variants of unknown significance (VUSs) were communicated after a multidisciplinary consultation where the clinical geneticist deemed the gene with the VUS matched (part of) the phenotype and the laboratory specialist found sufficient evidence for possible pathogenicity of the variant (i.e., allele frequency, conservation, *de novo*).

The incidental findings we reported fell into two categories: (1) (likely) pathogenic variants/CNVs predicted to cause an actionable disease, knowledge of which could lead to health benefits for the child and/or parents (8); (2) (likely) pathogenic variants in genes/CNVs associated with diseases unrelated to the phenotype, for which currently no therapy is available (i.e., developmental delay and/or intellectual disability).

### Study Outcomes

We measured diagnostic yield, TATs and the clinical consequences of the (rapid) genetic diagnostic approach. We distinguished diagnostic yield into three categories:

- Genetic diagnosis: genetic test result explains (most of) the phenotype
- Potential genetic diagnosis: reported VUS
- No diagnosis: no genetic cause was identified by genetic testing.

TAT was measured as the number of days between the first genetic consultation by the physician and the definitive report of genetic diagnostic results sent to the parents by the physician.

## Results

### Inclusion and Characteristics of Patients

Between December 2017 and January 2020, 171 infants received genetic consultation and 140 of these underwent genetic testing. Patients were retrospectively included in this study (see Table 1 for baseline characteristics). Most patients (73%) were admitted on first day of life (range 0–54 days) and most of the patients (98%) who were not admitted on the day of birth, had been admitted to a regional hospital before being transferred to the NICU of the UMCG. Of the 171 included patients, 26 have died during the 2-years study period: 21 patients died in the NICU and five patients died after discharge. The age at death ranged from 1 to 335 days, with a median of 22 days.

**Table 1:** Characteristics of 171 included patients.

**Primary organ system involved**	***N***	**%**
Multiple congenital anomalies	52	30.4
Cardiovascular	49	28.7
Gastro-intestinal	20	11.7
Suspected aneuploidy	17	9.9
Neurological	15	8.8
Musculoskeletal	9	5.3
Pulmonary	6	3.5
Urogenital	3	1.8
**Genetic testing**	140	81.9
Physicians declined testing	19	11.1
Parents declined testing	7	4.1
Other reasons	5	3.5

The most frequent reasons for referral for genetic consultation were the presence of multiple congenital anomalies involving at least two organ systems (30%, 52/171), congenital cardiovascular anomalies (29%, 49/171), followed by gastro-intestinal anomalies (12%, 20/171). The request for genetic consultation was made at a median age of 1 day of life (range 0–70 days), most often the day after admittance. For 19 patients, selected genetic testing had taken place prenatally. After birth, a second genetic consultation was requested if this was warranted by the postnatal phenotype of the patient. A total of 31 cases were excluded, because patients did not have genetic testing after consultation for the following reasons: (i) the parents declined testing, most commonly stating that genetic testing would not have an added value to them (*n* = 7); (ii) the clinical geneticists deemed genetic testing to be unnecessary as the condition was not likely to be caused by a genetic aberration (*n* = 19); (iii) the patient was transferred to a different University Medical Center (*n* = 3); (iv) a confirmed antenatal diagnosis (*n* = 1); and (v) insurance problems (*n* = 1) (see Table 1).

### Diagnostic Workflow: Phenotype-Based Sequencing vs. OMIM Morbid Gene Panel

**Figure 1:**
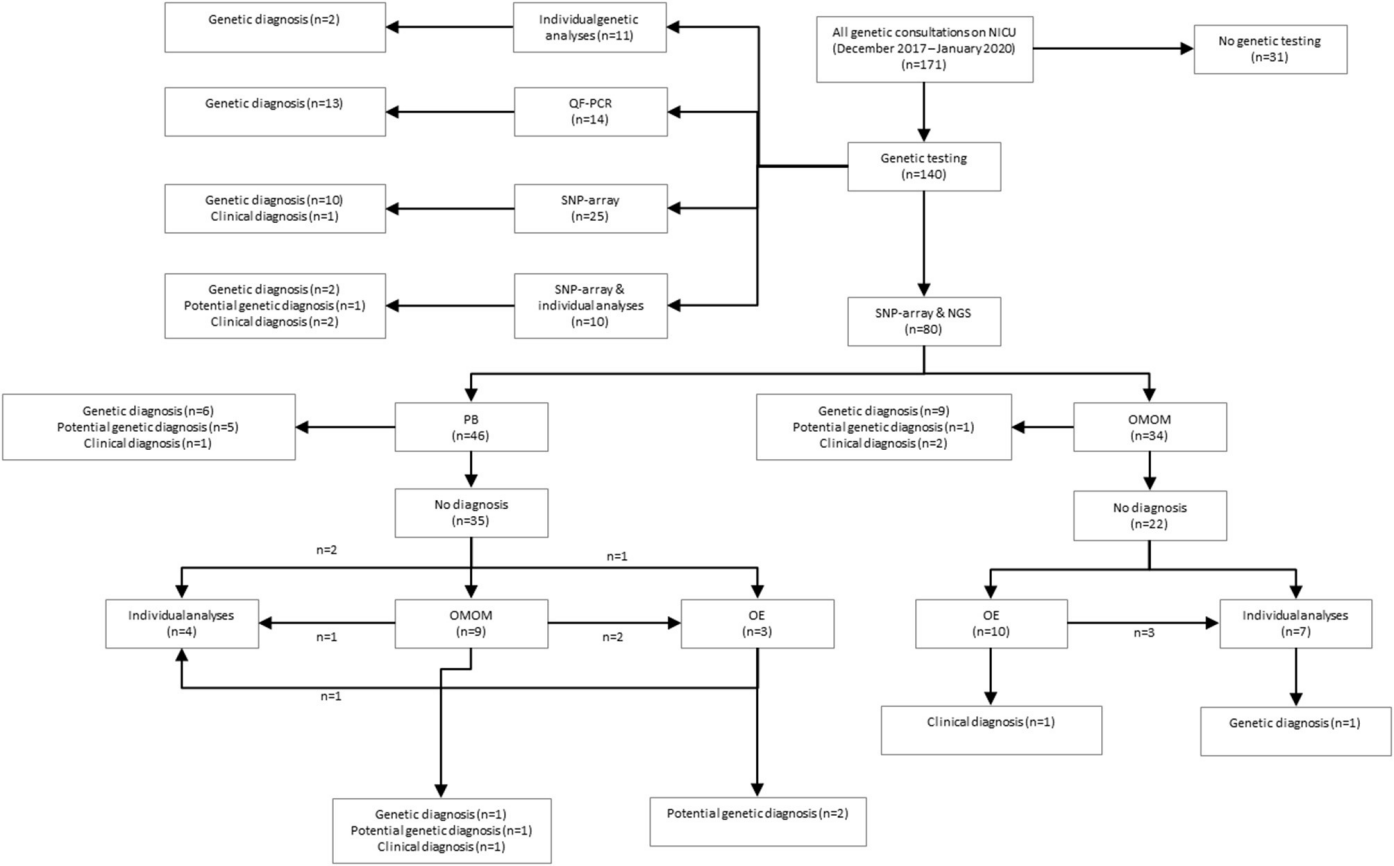
Schematic overview of study results. QF, quantitative fluorescent polymerase chain reaction; SNP, single nucleotide polymorphism array; NGS, next-generation sequencing; PB, phenotype-based targeted sequencing, OMOM, OMIM mendelian targeted sequencing; OE, open-exome analysis.

A total of 34 patients were offered SNP-array and the OMIM morbid gene panel as first tier diagnostic tests, because of the patient's complex phenotype. Nine out of these 29 patients were diagnosed: seven were diagnosed via the OMIM morbid gene panel and two showed a chromosomal abnormality with SNP-array. One patient was diagnosed after further testing, i.e., by analysis of the *DMPK* gene, associated with myotonic dystrophy, performed outside of the UMCG (a trinucleotide repeat as causal variant are not detected by ES). The median turnaround time till genetic diagnosis was 28 days (range 12–63 days) (Table 2). After OMIM morbid/SNP-array as first tier, 22 patients remained undiagnosed. Ten patients that could not be diagnosed by the OMIM morbid gene panel were offered open-exome analysis, but none were diagnosed with this technique (Figure 1). The 22 genetically undiagnosed patients had a median of three requested genetic tests (range 2–5). It took a median of 82 days (range 16–582 days) to complete all requested genetic tests.

**Table 2:** Diagnoses made with Next Generation Sequencing based tests.

**ID**	**M/F**	**Clinical features**	**Suspected diagnosis**	**Prenatal indication**	**Invasive prenatal diagnostics**	**Diagnostic techniques implemented**	**Genetic diagnosis**	**Clinical diagnosis**	**TAT (days)**	**Effect of diagnosis**
505	M	Congenital cardiomyopathy	LEOPARD syndrome, Bardet-Biedl syndrome, isolated cardiomyopathy	US		SNP, T (DCM)^*^	LP *MYH7*, AD, c.2711G>A, p.(Arg904His) *de novo*	Hypertrophic and dilating cardiomyopathy	28	Reproductive information for parents, screening family members for individual clinical risk
511	F	Congenital omphalocele, dysmorphic features	Beckwith-Wiedemann syndrome	US		SNP, T (CDKN1C), T (LIT1)^*^^&^	Hypomethylation of *LIT1*	Beckwith-Wiedemann syndrome	19	Reproductive information for parents, prognosis, follow-up advice according to published guidelines and/or recommendations
544	F	Respiratory distress, distinctive dysmorphic features	Treacher-Collins syndrome			T (HPO)^*^	P *POLR1D*, AD, c.259C>T, p.(Arg87^*^) pat	Treacher-Collins syndrome type 2	14	Reproductive information for parents, screening family members for genetic risk (offspring)
552	M	Polydactyly, bilateral cystic kidney malformation, dysmorphic features	Ciliopathy, cystic fibrosis			SNP, OMOM^*^^#^, T (*CFTR*)	P *HNF1B*, AD, c.541C>T, p.(Arg181^*^) pat	Renal cysts and diabetes syndrome	20	Reproductive information for parents, prognosis, follow-up advice according to published guidelines and/or recommendations
563	M	Cryptorchidism, clubfoot, dysmorphic features	Partial or mosaic trisomy 13/18, chromosomal or monogenic syndromes	US		SNP, OMOM^*^^#^	P *MYH3*, AD, c.533C>T, p.(Thr178Ile) *de novo*	Freeman-Sheldon syndrome	27	
574	M	Pulmonary valve stenosis, obstructive hypertrophic cardiomyopathy, cryptorchidism, dysmorphic features	Noonan syndrome, disorders of sex development	US		SNP, OMOM^*^^#^	P *RAF1*, AD, c.770C>T, p.(Ser257Leu) *de novo*	Noonan/LEOPARD syndrome	31	Reproductive information for parents
584	M	Arthrogryposis multiplex congenita, dysmorphic features	Amyoplasia congenita, distal arthrogryposis, fetal akinesia/ hypokinesia sequention, cerebro-oculo-facio-skeletal syndrome, chromosomal aberration, Escobar syndrome, trisomy 18	US		OMOM^*^^#^	P *ASCC1*, AR, c.710+1G>A	Spinal muscular atrophy (SMA) with congenital fractures type 2	38	Reproductive information for parents, prognosis, follow-up advice according to published guidelines and/or recommendations
610	F	Seizures, dysmorphic features	Neonatal convulsions/epileptic encephalopathy, chromosomal aberration			SNP, T (EPI/BFNC)^*^, T (EPI/EIEE)^*^	P *KCNQ3*, AD, c.988C>T, p.(Arg330Cys) *de novo*	(Benign) familial neonatal convulsions	17	Reproductive information for parents, prognosis, follow-up advice according to published guidelines and/or recommendations
617	M	Multiple abnormalities of the vertebrae, dysmorphic features	Spondylocostal dysostosis, VACTERL-association, CHARGE syndrome, Simpson-Golabi-Behmel syndrome, Goldenhar syndrome, chromosomal aberration	US		SNP, T (HPO), OMOM^*^	LP *PTPN11*, AD, c.1282G>A p.(Val428Met) *de novo*; VUS *ROBO2*, AD, c.3266C>T p.(Thr1089Met) *de novo*; VUS *TCF4*, AD, c.1319G>C p.(Gly440Ala) *de novo*	PTPN11-associated syndrome	108	Reproductive information for parents
619	F	Intestinal obstruction	Cystic fibrosis and pre-symptomatic testing for autosomal dominant polycystic kidney disease			T (*CFTR*), T (*PKD1*)^*^^$^	*PKD1*, AD, c.7376G>A p.(Gly2459Asp), pat	Autosomal dominant polycystic kidney disease	102	Reproductive information for parents
620	F	Cardiac malformation, dysmorphic features	No specific syndromic diagnosis suspected			SNP, OMOM^*^^#^	LP *NR2F2*, AD, c.1187T>A, p.(Ile396Asn) *de novo*	Heritable congenital heart defects, type 4	52	Reproductive information for parents, prognosis, follow-up advice according to published guidelines and/or recommendations
637	F	Hydrocolpos, hyperechogenic kidneys with cysts in the renal pyramids, dysmorphic features	HDR syndrome, McKusick-Kaufman syndrome, Bardet-Biedl syndrome	US		T (*GATA3*)^*^^@^	P *GATA3*, AD, c.404del p.(Pro135fs), mat	Hypoparathyroidism, sensorineural deafness and renal disease (HDR-syndrome)	87	Reproductive information for parents, prognosis, follow-up advice according to published guidelines and/or recommendations
659	M	Cardiac malformation, diaphragmatic hernia, absent gall bladder, vertebral and costal abnormalities, incomplete ossification of the sternum, dysmorphic features	Pentalogy of Cantrell			SNP, OMOM^*^	P *YY1*, AD, c.568_581del p.(Ala190Argfs^*^34) *de novo*	Gabriele de Vries syndrome	27	Reproductive information for parents, screening family members for individual clinical risk
671	F	Minor omphalocele, hypospadias, multiple sacral dimples, cryptorchidism, dysmorphic features	Beckwith-Wiedemann syndrome, Pit-Hopkins syndrome, glycogen storage disease, Fontaine progeroid syndrome	US	QF, SNP, OM	Re-analysis prenatal OMOM^*^, T (LIT1)^&^	P *CDKN1C* c.726dupG p.(His243Alafs^*^43) AD, mat	Beckwith-Wiedemann syndrome	63	Reproductive information for parents, prognosis, follow-up advice according to published guidelines and/or recommendations
673	M	Irregular tonus, retinal petechia, clubfeet, dysmorphic features	Chromosomal aberration, monogenic neuromuscular disease, monogenic metabolic disease, Ehlers-Danlos syndrome, clotting disease	US, NIPT		SNP, OMOM, T (*DMPK*)^*^^@^	3'UTR CTG(n>150) repeat *DMPK*	Myotonic dystrophy type 1	28	Reproductive information for parents, prognosis, follow-up advice according to published guidelines and/or recommendations

*TAT, Turnaround time; US, Abnormal ultrasound; NIPT, normal non-invasive prenatal test; QF, quantitative fluorescent polymerase chain reaction; SNP, single nucleotide polymorphism array; OMOM, OMIM mendelian targeted sequencing; T, phenotype-based targeted sequencing; DCM, with specific panel: dilated cardiomyopathy in children; CDKN1C, cyclin dependent kinase inhibitor 1C; LIT1, long QT intronic transcript 1; HPO, human phenotype ontology; CFTR, cystic fibrosis; EPI/BFNC, benign familial neonatal/infantile convulsions; EPI/EIEE, (Early Infantile) Epileptic Encephalopathy; PKD1, polycystin 1; GATA3, GATA binding protein 3; DMPK, DM1 protein kinase*;

(*) *, diagnosed using*;

(^#^) *, OM as first tier*;

(^&^) *, technique carried out in University Medical Centers Amsterdam*;

(^$^) *, technique carried out in Leiden University Medical Center*;

(^@^) *, technique carried out in University Medical Center Nijmegen; (mat), maternal; (pat); paternal; LP, likely pathogenic; P, pathogenic; AD, autosomal dominant; AR, autosomal recessive*.

A total of 46 patients were offered SNP-array and PB sequencing as first tier diagnostic techniques. Of these 46 patients, six were diagnosed: five were diagnosed using PB sequencing and one showed a chromosomal abnormality with SNP-array. For these six patients with a genetic diagnosis, a median of three (range two to four) genetic tests were requested. The median total turnaround time for these patients was 21 days (range 1–32 days) (Table 2). After PB sequencing/SNP-array as first tier, 35 patients remained undiagnosed. Of these, 26 patients had no further genetic testing, in 21 because the probable clinical diagnosis was a non-syndromic congenital heart defect and in five because features were concluded to be non-genetic (for instance post-ischemic or infectious). Nine patients who could not be diagnosed with this PB sequencing were offered the OMIM morbid gene panel, and of those nine, two patients were subsequently offered open-exome analysis. One patient was offered open-exome analysis immediately after PB sequencing. However, only one patient was diagnosed with one of these additional techniques (Figure 1). The 35 genetically undiagnosed patients had a median of two requested genetic tests (range 1–6). It took a median of 114 days (range 19–320 days) to complete all requested genetic testing.

To illustrate how TATs can become long, we describe one case in which subsequent phenotype-based analyses were requested. Patient 566 was born in a general hospital after an otherwise uncomplicated pregnancy. After birth, the patient was transferred to the UMCG, because of multiple congenital abnormalities. Genetic consultation was requested on the day of admittance to the NICU. Several clinical findings were reported, such as radial aplasia, multiple anomalies of the vertebra, esophagus atresia with fistula and dysmorphic features. Based on the clinical features, several syndromic causes were considered, including CHARGE syndrome, TAR syndrome, VACTERL-association, Nager syndrome and Treacher-Collins syndrome. SNP-array and the OMIM morbid gene panel tests were negative after 9 and 29 days, respectively. Open-exome analysis was requested for this patient and, after 126 days, no genetic cause could be detected. During this time it was also revealed that the patient suffered from an ASD/VSD. After this additional information it was concluded that the most probable diagnosis was VACTERL-association. The case was closed after 167 days.

### Diagnostic Yield

The overall yield of genetic testing in our cohort was 31% (44/140). QF-PCR and SNP array provided a diagnosis in 13/14 (93%) and 14/115 (12%) patients, respectively (Table 3). Phenotype-based sequencing provided a diagnosis in 4/46 (9%) patients, and the broader OMIM morbid gene panel provided a diagnosis in 9/43 (21%). Five patients (16%) were diagnosed after analysis of an individual gene such as *LIT1* and *GATA3* (Figure 1). After QF-PCR 10 patients were diagnosed with Down syndrome, two patients with Edwards syndrome and one with Patau syndrome.

**Table 3:** Diagnoses made with SNP-array.

**ID**	**M/F**	**Clinical features**	**Suspected diagnosis**	**Prenatal indication**	**Diagnostic techniques used**	**Genetic diagnosis**	**Clinical diagnosis**	**TAT (days)**	**Effect of diagnosis**
502	M	Esophagus atresia with TE-fistula, dysmorphic features	VACTERL- association, CHARGE syndrome, other syndromal cause		SNP^*^, T (CHARGE)^#^	1p36.23(8437274_8580322)x1 *de novo*	Causative *de novo* deletion	8	Reproductive information for parents, prognosis, follow-up advice according to published guidelines and/or recommendations screening family members for individual clinical risk
508	M	Dysmaturity, volvulus, ileal atresia, hypospadias, dysmorphic features	No specific syndromal suspicion		SNP^*^, OMOM	7q34q36.3(141536855_159138663)x1, 20p13p12.3(0_5745900)x3	Unbalanced translocation	16	Screening family members for genetic risk (offspring)
535	F	Respiratory distress, hypertrophic and dilated right ventricle, antenatal closure of ductus arteriosus, dysmorphic features	No specific syndromal suspicion	US	SNP	7q11.23(72722248_74185778)x1 *de novo*	Williams-Beuren syndrome	13	Reproductive information for parents, prognosis, follow-up advice according to published guidelines and/or recommendations
555	F	Atrial septal defect, ventricular septal defect, overriding aorta, dysmorphic features	Down syndrome	US	QF^*^, SNP^*^, T (PAH), T (*BMPR2*)	21q11.2q22.3(14359894_48129895)x3 *de novo*	Down syndrome	1	Reproductive information for parents, prognosis, follow-up advice according to published guidelines and/or recommendations
557	F	Perinatal asphyxia, trigonocephaly, dysmorphic features	Mother carrier of balanced translocation chromosome 1 and 9 (46,XX,t(1,9)(q23.3;p.23))	US	SNP	1q32.3q44(212955231_249218992)x3, 9p24.3p23(46587_13806091)x1 mat	Unbalanced translocation	6	Reproductive information for parents, palliative care
571	F	Cardiac malformation, dysmorphic features	Turner syndrome with additional chromosomal or monogenetic aberration	US	SNP	(X)x1	Turner syndrome	16	Reproductive information for parents, prognosis, follow-up advice according to published guidelines and/or recommendations
576	M	Central hypotonia, possible myoclonic seizures, cryptorchidism, dysmorphic features	Chromosomal or Monogenetic syndromes, i.e., Prader-Willi syndrome or PHG6, VPS13B		SNP	15q11.2q13.1(23677677_28830871)x1 *de novo*	Prader-Willi syndrome	22	Reproductive information for parents, prognosis, follow-up advice according to published guidelines and/or recommendations, treatment, specific advice
577	M	Morbus Hirschsprung, asymmetric crying face, ventricular septal defect, dysmorphic features	22q11 deletion		QF^*^, SNP^*^	22q11.21(18687210_21644673)x1 *de novo*	22q11.2 deletion syndrome	8	Reproductive information for parents, prognosis, follow-up advice according to published guidelines and/or recommendations, treatment, specific advice
592	F	Truncus arteriosus, dysmorphic features	22q11 deletion	US	QF^*^, SNP^*^	22q11.21(18876616_21644673)x1 *de novo*	22q11.2 deletion syndrome	3	Reproductive information for parents, prognosis, follow-up advice according to published guidelines and/or recommendations, treatment, specific advice
599	M	Ventricular septal defect, dysmorphic features	Syndromal cause	US	SNP	9q34.3(138683334_141213431)x1 *de novo*	Kleefstra syndrome	16	Reproductive information for parents, prognosis, follow-up advice according to published guidelines and/or recommendations
605	M	Macrosomia, anal atresia, cardiac malformation, bilateral hydronephrosis, dysmorphic features	VACTERL-association, cat-eye syndrome, chromosomal aberration		SNP^*^, T (HT)	20p11.22p11.21(22054764_24364361)x1 *de novo*	Causative *de novo* deletion	11	Prognosis, follow-up advice according to published guidelines and/or recommendations, screening family members for genetic risk (offspring)
607	F	Cardiac malformation, dysmorphic features	Kleefstra syndrome, Turner syndrome	US	SNP	Yq12(154,934,000-155,270,560)x1, 9q34.3(138,083,055-141,213,431)x1 pat	Kleefstra syndrome	12	Prognosis, follow-up advice according to published guidelines and/or recommendations
635	M	Kidney malformation, abnormal gall bladder, haemangioma of the liver, splenic cysts, dysmorphic features	Polycystic kidney disease, ciliopathy, Pallister-Killian syndrome	US	SNP^*^, T (CP)^&^	22q11.21(18886915_21463730)x3 *de novo*	Atypical 22q11.2 duplication syndrome	26	Prognosis, follow-up advice according to published guidelines and/or recommendations
641	F	Polycystic dysplastic right kidney, cardiac malformation	22q11.2 deletion syndrome, Kleefstra syndrome, ciliopathy, Kabuki syndrome	US	SNP^*^, OMOM	16p12.2(21827582_22425152)x1 *de novo*	16p12.1 deletion syndrome	12	Reproductive information for parents, prognosis, follow-up advice according to published guidelines and/or recommendations, screening family members for individual clinical risk

*TAT, Turnaround time; PPHN, Persistent pulmonary hypertension of the newborn; US, abnormal ultrasound; QF, quantitative fluorescent polymerase chain reaction; SNP, single nucleotide polymorphism array; OMOM, OMIM mendelian targeted sequencing; PB, phenotype-based targeted sequencing; CHARGE, with specific panel: CHARGE-syndrome; CCHS, congenital central hypoventilation syndrome; PAH, pulmonary arterial hypertension; BMPR2, bone morphogenetic protein receptor type II; HT, heterotaxia; CP, ciliopathy*;

(*) *, diagnosed using*;

(^#^) *, technique carried out in University Medical Center Nijmegen*;

(^&^) *, technique carried out in University Medical Center Utrecht; (mat), maternal; (pat), paternal*.

In addition to (likely) pathogenic variants, to ten patients, one or multiple VUS(s) were reported as the potential genetic diagnosis. Including these potential genetic diagnoses, the diagnostic yield was 54/140 (39%). VUSs were reported after PB sequencing to five patients, two after the OMIM morbid gene panel, two after open-exome analysis and one after exome sequencing in another academic center (Table 4).

**Table 4:** Reported variants of unknown significance.

**ID**	**M/F**	**Clinical features**	**Suspected diagnosis**	**Prenatal indication**	**Invasive prenatal diagnostics**	**Diagnostic techniques implemented**	**Potential genetic diagnosis**	**Clinical diagnosis**	**TAT (days)**	**Effect of diagnosis**
532	F	Hyperekplexia, dysmorphic features	Monogenic disease			SNP, T (CCHS), T (HPO), T (MYO), OMOM, OE^*^	VUS *WDR47*, AD, c.2392C>G, p.(Arg798Gly), *de novo*; VUS *KCNH5*, AD, c.1388T>C, p.(Ile463Thr), *de novo*	KCNH5-associated hyperekplexia	201	Reproductive information for parents
567	M	Respiratory distress, hypotonia, abnormal aortic anatomy, convulsions, dysmorphic features	Oculo-auriculo-vertebral spectrum, aortic arch anomalies, mosaic trisomy 8			SNP, T (*MECP2*), T (CCHS), OMOM, OE^*^	VUS *GSPT2*, AD, c.872A>G, p.(His291Arg), mat	GSPT2-associated syndrome	29	Reproductive information for parents
575	M	Bilateral choanal atresia, dysmorphic features	CHARGE syndrome, chromosomal aberration, syndromal disorder			SNP, ES^*^^@^	VUS *KMT2D*, AD, c.10658G>T p.(Gly3553Val), *de novo*	KMT2D-associated syndrome	282	Reproductive information for parents, prognosis, follow-up advice according to published guidelines and/or recommendations
583^%^	F	Hydrops fetalis, dysmorphic features	Autosomal recessive disorder, chromosomal aberration, Noonan syndrome	US		SNP, OMOM^*^^#^, OE	VUS *GDF2*, AR, c.451C>T, p.(Arg151^*^), pat/mat	GDF2 related hydrops	16	Reproductive information for parents, prognosis, follow-up advice according to published guidelines and/or recommendations
612	M	Pulmonary atresia, ventricular septal defect, overriding aorta, dysmaturity, glandular hypospadias, dysmorphic features	No specific syndromal suspicion	US	QF, SNP	T (CHD)^*^	VUS *POGZ*, AD, c.3761C>T, p.(Pro1254Leu), *de novo*	White-Sutton syndrome	32	Prognosis, follow-up advice according to published guidelines and/or recommendations, screening family members for individual clinical risk
623	F	Pulmonary atresia with intact ventricle septum, icterus, dysmorphic features	Monogenic disease, chromosomal aberration			SNP, T (CHD)^*^, T (MDL)	VUS *MYH6*, AD, c.5129A>T, p.(Glu1710Val), pat	MYH6-related cardiac defect	23	Reproductive information for parents, prognosis, follow-up advice according to published guidelines and/or recommendations, screening family members for individual clinical risk
624	M	Dandy Walker malformation, cerebellar cyst, dysmorphic features	Ciliopathy, G-syndrome, Smith-Lemli-Opitz syndrome, Coffin-Siris syndrome, Galloway-Mowat syndrome, Walker-Warburg syndrome	US	QF, SNP	T (NEURO), OMOM^*^	VUS *CDC42*, AD, c.353A>G p.(Asp118Gly), *de novo*	Takenouchi-Kasaki syndrome	57	Reproductive information for parents
625	F	Flexion stance of the digits, dysmorphic features	Distal arthrogryposis type 1 or type 2, amyoplasia, Freeman-Sheldon syndrome, Gordon syndrome, monogenic disease			SNP^*^, T (HPO)^*^	10q11.22q11.23(49341473_51077802)x1 mat, 10q11.23(51781571_52434258)x1, mat; VUS *SLC18A3*, AR, c.1163G>A, p.(Cys388Tyr), pat	Presynaptic congenital myasthenia	28	Reproductive information for parents, prognosis, follow-up advice according to published guidelines and/or recommendations, screening family members for individual clinical risk
629	M	Neonatal seizures, dysmorphic features	Benign familiar neonatal convulsions, epileptic encephalopathy			SNP, T (EPI/BFNC)^*^, T (EPI/EIEE)^*^, T (EPI/BNFC)^*^^&^	VUS *KCNQ2*, AD, c.1735C>G, p.(Leu579Val), mat	(Benign) neonatal epileptic encephalopathy	22	Reproductive information for parents, palliative care
655	M	Cardiac malformation, dysmorphic features	Monogenic disease, syndromal disorder	US	QF, SNP	T (CHD)^*^	VUS *LZTR1*, AD, c.1165C>T p.(Leu389Phe), *de novo*	Noonan syndrome	20	Reproductive information for parents, prognosis, follow-up advice according to published guidelines and/or recommendations

*TAT, Turnaround time; US, Abnormal ultrasound; QF, quantitative fluorescent polymerase chain reaction; SNP, single nucleotide polymorphism array; OMOM, OMIM mendelian targeted sequencing; OE, open-exome analysis; ES, Exome sequencing; T, phenotype-based targeted sequencing; HPO, with specific panel: human phenotype ontology; MYO, myoclonic epilepsy; CCHS, congenital central hypoventilation syndrome; MEPC2, methyl-CpG-binding protein; CHD, congenital heart disease; MDL, cholestasis/acute liver failure; NEURO, Joubert-like conditions; EPI/BFNC, benign familial neonatal/infantile convulsions; EPI/EIEE, (Early Infantile) epileptic encephalopathy*;

(*) *, diagnosed using*;

(^#^) *, OM as first tier*;

(^@^) *, technique carried out in University Medical Center Nijmegen*;

(^&^) *, technique carried out in University Medical Center Utrecht; mat, maternal; pat), paternal; LP, likely pathogenic; VUS, variant of unknown significance; AD, autosomal dominant; AR, autosomal recessive; (%), recently published as case report by Aukema et al. (18)*.

### Incidental Findings

Incidental findings (IFs) were found in six out of 140 tested patients (4%). Of these, three were identified in 43 OMIM morbid panel analyses (7%) and three in 115 SNP-arrays (3%). Two of the IF's in OMIM morbid gene panels were actionable IFs (*MYH7, PSKH9*) in accordance with the ACMG (8). No IF's were found with QF, PB sequencing, open-exome analysis or individual gene analysis (Table 5).

**Table 5:** Reported incidental findings.

**IF category**	**Diagnostic techniques implemented**	**Incidental finding**	**Consequence**
Variant(s) matching inheritance pattern of the actionable disease	OMOM	P *COL6A3*, AD/AR, c.6354+2T>C, *de novo*	(Ulrich congenital) muscular myopathy/Bethlehem myopathy
	OMOM	^*^LP *MYH7*, AD, c.5326A>G, p.(Ser1776Gly), pat	Hypertrophic cardiomyopathy
	OMOM	^*^P *PCSK9*, AD, c.1394C>T p.(Ser465Leu), mat	Hypercholesterolemia
	SNP	15q11.2(22766393_23272175)x1, mat	15q11.2 microdeletion syndrome
	SNP	17q12(34652993_36428544)x3, *de novo*	17q12 microduplication syndrome;
	SNP	(X)x2, (Y)x1, *de novo*	Klinefelter syndrome

*SNP, Single nucleotide polymorphism array; OMOM, OMIM morbid gene panel sequencing; mat, maternal; pat, paternal; P, pathogenic; LP, likely pathogenic; AD, autosomal dominant; AR, autosomal recessive*;

(*) *, actionable according to ACMG (8)*.

## Discussion

The results of our retrospective study demonstrate that a broad gene panel including all OMIM Mendelian genes is the favorable exome sequencing approach in rapid genetic diagnostics in neonatal intensive care. This broad OMIM morbid gene panel resulted in a higher diagnostic yield than a dedicated phenotype-based gene panel, namely a 21 vs. 9% yield. The TATs for both as primary test were comparable. The most probable explanation for the low diagnostic yield using a targeted panel is that we are not as good as we like to believe in making clinical diagnoses in this group of patients: most patients in the NICU have complex phenotypes, which are not easily recognized early after birth, a problem which is aggravated by the limitations of physical examination in a critical care setting.

The overall diagnostic yield of genetic testing in our cohort was 31%, but this differed per test. CNV detection using SNP-array and chromosome number anomaly detection using QF-PCR resulted in the most diagnoses, 14 and 13, respectively (32 and 30% of the diagnostic yield, respectively), followed by the broad OMIM morbid gene panel, with nine diagnoses (20% of the diagnostic yield). Comparison with other studies is difficult because the inclusion criteria, numbers of patients and data analyses are different in each study. A large cohort of 129 patients showed 20% diagnostic yield, regardless of rapid ES or rapid WGS, and found 25 SNV/indel (13%), 10 CNV (6%), and two aneuploidy (1%) diagnoses (4). In the RAPIDOMICS approach (19), which included 25 patients, the diagnostic yield was 72% using trio analyses and an OMIM based gene panel. In a comparable study, the diagnostic yield was 50% in 20 patients (20). A multi-center based Australian study included 108 neonatal and pediatric ICU patients and achieved a diagnostic yield of 51% (21). Another Dutch study reported an overall genetic diagnostic yield of 24.2% in 132 patients from the NICU/PICU/medium neonatal care units; of these 132 patients, 31 patients had an exome sequencing test with a yield of 17% (22). In general, the diagnostic yield of rNGS in NICU care is strongly dependent on inclusion criteria and genetic tests performed before.

In addition to (likely) pathogenic variants, VUSs were reported to patients in 10 cases. These are variants in genes that match the phenotypic features of the patient, but for which there is not enough current evidence to classify them as likely pathogenic. Reporting these strongly suggestive variants allows for follow-up studies. Looking for other patients in Genematcher (23), segregation studies, or functional studies might help to re-classify such VUSs.

Based on our results, we suggest QF-PCR should be performed as first tier only when phenotypic features are strongly suggestive of trisomy 13, 18, or 21, and no NIPT has been performed during pregnancy, as QF-PCR is very fast and cheap. Exome sequencing should be performed with a broad gene panel for SNV and CNV detection or a separate array as CNV test. Because of the limited yield with single gene testing or a dedicated PB virtual panel, these tests are less preferable with the patient population present on the NICU. This is supported by the large number of patients (*n* = 9; 26%) where a targeted panel had to be followed up by the OMIM morbid gene panel. The ones that did not proceed to further testing were mainly patients with single organ anomalies (for instance congenital heart malformations). Dillon et al. even reported that using a panel may lead to missing diagnoses: in their study 23% of exome sequencing-diagnosed children would not have been diagnosed had a panel been selected (24). Xue et al. published a genetic testing algorithm for Mendelian disorders in which they distinguish between distinctive clinical features followed by single gene/panel testing vs. multiple non-specific concerns followed by array and exome sequencing trio testing (25), and our diagnostic yield per test supports this suggested workflow. However, Xue et al. do not discuss a large OMIM morbid gene panel as an intermediate between PB and exome sequencing (in which all ~23,000 genes are assessed). Moreover, it is important to keep in mind that it might not be as easy to define distinct clinical features in the special group of NICU patients.

A straightforward approach of starting with a broad gene panel, not biased by the phenotype interpretation, can reduce the total TAT because it makes sequential testing obsolete. Although the TAT for the targeted small PB panel approach and the broad OMIM morbid gene panel approach as primary test was comparable (14 days on average), the TAT for a diagnosis was much longer for the group of patients with a PB panel as initial test, due to required further testing. Because a genetic diagnosis was made in only four cases in this scenario, and other PB panels and/or an OMIM morbid gene panel were subsequently requested. This meant that total TAT until diagnosis for these cases was a median of 114 days, on average, and up to 320 days. Focusing on one OMIM morbid gene panel as primary test allows for further automation in sample handling and data interpretation, which can further reduce TATs. In our laboratory, we incorporated additional filtering based on a variant's mode of inheritance in the patient into our OMIM morbid gene panel filtering strategy, which reduced interpretation time, thereby reducing the number of variants for interpretation.

A drawback of testing a large set of genes might be the increased chance of incidental findings (IFs). In our study we found eight IFs in total, of which five were found with the OMIM morbid gene panel and three were found with SNP-array. In accordance with current Dutch guidelines for IFs, carriage of a (likely) pathogenic variant in an autosomal recessive gene are not reported. In the pretest counseling, parents are informed about IFs. Opting out for IFs associated with diseases with therapeutic options during childhood is not offered to parents. Given the higher number of genetic diagnoses using the OMIM morbid gene panel, and the only slightly increased number of actionable IFs, we think testing using a broad gene panel in NICU patients is justified. However, strategies for data filtering should be focused on not detecting IFs, for instance by filtering late onset diseases in the setting of diagnostics for infants.

The benefit of an extra open-exome approach is that it allows for the detection of novel genes and for associating known genes with complex phenotypes. However, this approach depends on the diligence of all clinical staff in keeping up with publications and contacting colleagues with patients with similar phenotypes or with variants in the same genes (data-sharing), and this is an investment that many diagnostic laboratories are not able to make. To alleviate this problem, the latest generation of bioinformatics tools may offer complementary approaches to identify potentially causal variants located outside of currently known disease genes. For instance, a prediction of clinical relevance may be assigned to unknown genes using algorithms that perform gene prioritization based on patient symptoms, provided that the tool of choice covers at least a majority of all expressed genes in the genome (26). To discover the most promising variants within these prioritized unknown genes, the latest genome-wide variant pathogenicity estimates (27) could be utilized in combination with gene-specific probabilities of intolerance to loss-of-function and missense mutations (12). Labor-intensive functional follow ups and contacting of colleagues can then be focused on only the most promising candidates, directing precious resources to those variants of which the chances of reaching a final molecular diagnosis are the highest.

In conclusion, analyzing a broad gene panel in parallel with CNV testing, ideally within one test, as the first tier of genetic testing for NICU patients is the best choice to identify the genetic cause of their condition quickly. However, data filtering strategies are necessary to minimize the chance of detecting IFs as much as possible, and informed consent of the parents and a clear policy/protocol how to handle IFs are obligatory. To shorten TATs, effort should be put into further automation of laboratory procedures and data interpretation. Careful description of phenotypic features and monitoring and reporting of VUSs that likely explain the phenotype are essential to pinpoint to novel disease-causing genes, while data sharing, segregation analyses and functional testing now need priority to re-classify these VUSs.

## Data Availability Statement

The data analyzed in this study is subject to the following licenses/restrictions: The dataset is generated in a hospital setting as diagnostics. The patient data are not allowed to leave the hospital's secured server environment. Requests to access these datasets should be directed to Cleo C. van Diemen, c.c.van.diemen@umcg.nl.

## Ethics Statement

The studies involving human participants were reviewed and approved by Medisch Ethische Toetsingscommissie University Medical Center Groningen. Written informed consent to participate in this study was provided by the participants' legal guardian/next of kin.

## Author Contributions

MI retrieved the clinical and genetic data, performed the data analysis, and drafted the manuscript. BS-R, WK-F, and CD contributed to the conception and design of the research, contributed to interpretation of the data, and co-wrote the manuscript. KA, MM-V, MS, KV, GB, DB, and NK contributed to clinical and genetic testing data collection, analysis, and interpretation of the data. All authors critically revised the manuscript and read and approved the final manuscript.

## Conflict of Interest

The authors declare that the research was conducted in the absence of any commercial or financial relationships that could be construed as a potential conflict of interest.
